# Multicenter Study of Clinical Presentation, Treatment, and Outcome in 41 Dogs With Spinal Epidural Empyema

**DOI:** 10.3389/fvets.2022.813316

**Published:** 2022-03-07

**Authors:** Emma J. Laws, Lluís Sánchez, Elsa Beltran, Elisabet Domínguez, Abel B. Ekiri, Josep Brocal, Luisa De Risio

**Affiliations:** ^1^Linnaeus Veterinary Limited, Solihull, United Kingdom; ^2^Neurology/Neurosurgery Service, Willows Veterinary Centre and Referral Centre, Linnaeus Veterinary Limited, Solihull, United Kingdom; ^3^Neurology/Neurosurgery Service, Queen Mother Hospital for Animals, Royal Veterinary College, Potters Bar, United Kingdom; ^4^Diagnostic Imaging Service, Anicura ARS Hospital Veterinari, Barcelona, Spain; ^5^Department of Veterinary Epidemiology and Public Health, School of Veterinary Medicine, University of Surrey, Guildford, United Kingdom; ^6^Neurology/Neurosurgery Service, Anderson Moores, Linnaeus Veterinary Limited, Winchester, United Kingdom

**Keywords:** empyema, infection, dog, spinal epidural empyema, spinal epidural abscess, epidural

## Abstract

There is limited information on canine spinal epidural empyema (SEE). The aim of this multicenter retrospective study is to describe the clinical presentation and outcome of dogs undergoing spinal surgery or conservative management for SEE. Forty-one dogs met the inclusion criteria; the SEE was treated surgically in 17 dogs and conservatively in 24 dogs. Two dogs underwent spinal surgery after failure of conservative management, meaning that 19 dogs in total had spinal surgery. Long-term (i.e., >6 months) follow-up was available in 35 dogs (19 conservatively treated and 16 surgically treated dogs). Recovery to a functional pet status was achieved in 15/19 (78.9%) conservatively treated and 12/16 (75%) surgically treated dogs. There was no significant difference (*p* = 1.000) in long-term outcome between conservatively and surgically treated dogs (78.9 and 75%, respectively). However, significantly more surgically treated dogs were non-ambulatory at presentation (9/17 vs. 5/24, *p* = 0.048) compared with conservatively treated dogs. This study suggests that conservative treatment may be appropriate for dogs with SEE that are ambulatory at presentation and that surgically treated dogs generally have good outcomes. Age may be a negative prognostic indicator as dogs with poor long-term outcomes were significantly older than dogs with a good long-term outcome (*p* = 0.048). A larger prospective randomized study may provide further insight on treatment and outcome of SEE in dogs.

## Introduction

Spinal epidural empyema (SEE) is defined as the accumulation of purulent material in the epidural space of the vertebral canal (1). It can affect humans (2), dogs (3–7), and other species (8–13) leading to neurological disability and mortality (5).

Microorganisms enter the epidural space *via* hematogenous spread (2, 4, 14, 15), direct extension (2, 3, 5–7, 15–24), iatrogenic inoculation (2, 25), or trauma (2, 6, 15). Suggested pathophysiological mechanisms to explain neuronal damage include ischemia from compression or disruption of vascular supply secondary to septic thrombophlebitis and secondary inflammation of neuroparenchyma itself, in particular in cases where the LS region is affected (e.g., secondary inflammation of the cauda equina) (26). Neurological symptoms and signs reported in human and canine SEE include spinal pain and progressive neurological dysfunction, including paresis, plegia, and incontinence (2, 5).

SEE can be treated surgically or conservatively (15, 27). Surgical treatment involves epidural pus drainage and appropriate antimicrobial therapy, while conservative treatment consists of adequate antibiotherapy (15, 27). In the human literature, it is unclear whether patients undergoing surgical drainage of paraspinal abscesses without direct spinal cord and/or cauda equina decompression are considered to be conservatively or surgically treated.

The choice between conservative and surgical management in humans remains controversial (28–31). Most human studies recommend that if surgery is performed, it is done within 24 h of diagnosis (28, 30, 32–35). However, conservative management can be successful (27, 28, 30, 33, 36–38), particularly in patients without neurological deficits (30, 35, 39, 40). In canine SEE, it is unknown whether superior clinical outcomes are achieved with conservative or surgical treatment. Most veterinary reports of SEE are single cases, with the largest case series comprising seven dogs (5). Of the 39 previously published canine SEE cases, 12 were managed conservatively (4, 15, 25, 41–43), 24 surgically (5–7, 14, 16–23), and 3 were not treated (3, 4, 24). Of the 12 conservatively treated dogs, only 2 had a poor outcome and were euthanized following respiratory arrest (4) and neurological deterioration (43). One of these two dogs also had intracranial involvement (4). Of the 24 surgically managed dogs, 4 had a poor outcome (5–7); however, one was euthanized without further investigations after relapse of severe spinal pain 1 week post-surgery (6), and one was euthanized due to an unrelated cervical intervertebral disc extrusion 1 month postoperatively (5). For the remaining two dogs, one was euthanized due to worsening neurological signs and pneumonia (5), and one died after cardiac arrest in the immediate postoperative period (7).

The aim of this study is to describe the clinical presentation and outcomes of conservatively and surgically treated dogs with SEE that presented to five referral hospitals. We hypothesized that dogs that presented as non-ambulatory were more likely to be treated surgically and that conservative treatment may be appropriate for dogs without neurological deficits.

## Materials and Methods

### Study Sites

The clinical database of five UK veterinary referral centers was searched for dogs diagnosed with SEE. The time period searched varied depending on the availability of a neurology service and high-field MRI in each included institution: January 1, 2000–December 31, 2020 at the first institution; January 1, 2000–December 31, 2019 at the second institution; January 1, 2009–December 31, 2020 at the third institution; January 1, 2015–December 31, 2020 at both the fourth and fifth institutions.

### Inclusion Criteria

The inclusion criteria were a diagnosis of SEE based on clinical presentations, MRI findings consistent with previously reported MRI features of SEE (6, 20, 23), and/or surgically confirmed SEE. Previously reported MRI features of SEE that were used included T2W high/mixed signal and T1W low signal epidural mass lesion with concurrent T2W hyperintensity within spinal cord gray matter (6, 20, 23). Only dogs that had undergone 1.5 T MRI were included. Each MRI study was reviewed by the same board-certified radiologist (ED) at the time of the study to further validate the diagnosis of SEE and to carefully evaluate all the MRI findings detailed in the MRI section that follows. Dogs with concomitant intracranial involvement were excluded.

### Data Collection

The following data were obtained from the medical records and recorded in an Excel document: signalment, clinical signs observed by the dog owners, pre-referral treatment, general physical and neurological examination findings (at the referral centers), concurrent conditions, time from the onset of clinical signs to SEE diagnosis, laboratory and MRI findings, and details of treatment and outcome.

Data on laboratory investigations included hematology, serum biochemistry, urine and blood cultures, cerebrospinal fluid (CSF) analysis and culture, and histopathological examination and culture of epidural material (in surgically treated dogs). Anemia was classified as either mild [hematocrit (HCT): 0.30–0.36], moderate (HCT: 0.18–0.29), or severe (HCT: <0.18).

Data on high-field (1.5 T) MRI images of the vertebral column were collected for all enrolled dogs, which were reviewed by a board-certified diagnostic imaging specialist (ED). The regions of the vertebral column undergoing MRI were determined based on the neuro-anatomic localization following neurologic examination by a board-certified veterinary neurologist. MRI scanners at the different referral institutions were Signa EchoSpeed (GE Healthcare, Milwaukee, Wisconsin, USA), Intera (Philips Healthcare, Eindhoven, The Netherlands), Vantage ELAN (Canon Medical Systems, Tustin, California, USA), and Magnetom Essenza Dot (Siemens Healthcare, Munich, Germany).

T2-weighted (TR range 2,541–13,681 ms; TE range 81–120 ms) images in the transverse, sagittal, and/or dorsal planes were obtained for all dogs. T1-weighted (400–660 ms; 8–16 ms) (T1W), T1W fat saturated (8–620 ms; 4–15 ms) after intravenous gadolinium (0.1 mmol/kg gadobutrol, Gadovist; Bayer) administration, T2^*^ gradient echo (300–440 ms; 9–15 ms), short tau inversion recovery (3,420–4,270 ms; 13–80 ms), and fluid-attenuated inversion recovery (6,000–8,002 ms; 98–120 ms) images were obtained at the discretion of the attending diagnostic imaging specialist and neurologist. The slice thickness was 1–10 mm. The following MRI features were recorded: SEE location (cervical, i.e., C1–C7 vertebral bodies; thoracic, i.e., T1–T13 vertebral bodies; and/or lumbosacral, i.e., L1–S3 vertebral bodies and cauda equina), the presence/absence of abnormal intramedullary signal, the pattern of epidural material contrast enhancement, the presence of changes compatible with discospondylitis, and the presence of extra-spinal foci of suspected inflammation or infection.

The pre- and post-treatment neurological status was scored using a six-point grading scale [adapted from Scott (44)] based on the information detailed in the neurologic examination form of each dog (at the referral hospital): grade 0 (neurologically normal), 1 (pain without neurological deficits), 2 (ambulatory paresis), 3 (non-ambulatory paresis), 4 (plegia with nociception), or 5 (plegia without nociception). Dogs exhibiting a stiff gait and/or lameness without any further neurological deficits were classified as grade 1. Urinary and fecal continence were assessed separately.

The decision to treat SEE conservatively or surgically was at the discretion of the attending neurologist and dog's owner. Dogs were divided into two groups: dogs treated with spinal cord decompression plus antibiotic therapy (referred in this article as spinal surgical treatment group or surgically treated dogs), and dogs treated with antibiotics alone or combined with abdominal surgery to debride a paraspinal abscess (referred in this article as conservative treatment group or conservatively treated dogs). All dogs received anti-inflammatories [non-steroidal anti-inflammatory drugs (NSAIDs) or glucocorticoids] and analgesia (opioids, lidocaine, gabapentin, paracetamol, or ketamine).

Information regarding complications during hospitalization and duration of hospitalization was retrieved from the medical records. Neurological grade was determined at the time of discharge and at re-examination at the referral hospital 4–8 weeks post-discharge. Long-term follow-up was defined as a follow-up period of at least 6 months and was obtained *via* the referral centers' or referring practices' medical records and/or postal questionnaires sent to the dogs' owners. The outcome was considered successful if the dog was a functional pet (independently ambulatory, urinary and fecally continent, and considered by their owner to be pain-free with a good quality of life). The outcome was considered poor if the dog was euthanized or died due to SEE, was euthanized or died due to systemic disease potentially associated with SEE, or if it had not become a functional pet by ≥6 months post-discharge.

### Statistical Analysis

Descriptive statistics were used to compare clinical presentation and outcome variables between the treatment groups. Fisher's exact test was used to compare categorical data, Mann–Whitney test to compare ordinal data, and two-sample *t*-test for continuous data (after checking for normality using the Shapiro–Wilk test). For all analyses, a two-sided *p*-value of < 0.05 was considered significant, and all analyses were performed by a biostatistician using STATA (version 14.2; StataCorp, College Station, Texas, USA).

## Results

Forty-one dogs met the inclusion criteria: 24 were initially treated conservatively (including 3 who underwent surgical debridement of a paraspinal abscess without entering the vertebral canal) and 17 underwent decompressive spinal surgery (e.g., minihemilaminectomy, hemilaminectomy, or dorsal laminectomy and debridement of the empyema) shortly after MRI. Two dogs underwent spinal decompressive surgery after failure of conservative management; therefore, the total number of dogs undergoing spinal surgery was 19. These two dogs are included in the conservatively treated group for signalment and clinical presentation, location, and MRI findings, while in the surgical group for neurological status at discharge and outcome.

### Signalment and Clinical Presentation

Median age at presentation was 6 years (range, 0.3–13.3years) for all 41 dogs included in the study, 7 years (range, 0.3–13.3 years) in the 24 dogs who underwent conservative treatment, and 4 years (range, 3–11 years) in the 17 dogs that underwent spinal surgery for SEE. There was no significant difference (*p* = 0.298) in age at presentation between the conservatively and surgically managed groups. Twenty-six dogs were male (17 conservatively treated and 9 surgically treated) and 15 dogs were female (7 conservatively treated and 8 surgically treated). Twenty-one dogs (51%) were entire [16/26 male dogs (61.5%) and 5/19 (26.3%) female dogs]. The most commonly represented breeds included springer spaniels (*n* = 7), rottweilers (*n* = 4), German shepherds (*n* = 4), border collies (*n* = 3), boxers (*n* = 3), crossbreeds (*n* = 2), bull terriers (*n* = 2), English bulldogs (*n* = 2), and French bulldogs (*n* = 2).

In 40/41 (97.5%) dogs, pain, lameness, and a stiff gait were the first neurological signs observed by the dog owners. Other owners reported clinical signs (in the 24 conservatively treated and 17 dogs who underwent spinal surgery, respectively) included lethargy (13/24 and 10/17 dogs), hyporexia (7/24 and 6/17 dogs), paresis (8/24 and 4/17 dogs), diarrhea (3/24 and 1/17 dogs), respiratory distress (2/24 and 1/17 dogs), and weight loss (2/24 and 1/17 dogs). In addition, 1 dog exhibited vomiting.

Information on pre-referral medications was available in 38 dogs (21 conservatively treated and 17 surgically treated dogs). Pre-referral antibiotics were prescribed to 8/21 conservatively treated and 7/17 surgically treated dogs. Pre-referral analgesia was prescribed to all 38 dogs with information available. Corticosteroids at varying doses had been administered to four dogs for non–SEE-related conditions. Three of these dogs had courses >12 months in duration, and one dog had a course for 1 month prior to referral. Clinical signs detected at the time of initial examination at the referral hospital are summarized in Table 1. The only significant finding was that more dogs in the conservatively treated group were ambulatory compared with the surgically treated group (*p* = 0.048). Other than the ambulatory status, there were no other significant differences in clinical presentation parameters between the two groups.

**Table 1:** Clinical signs detected at the time of presentation to the referral hospital of 41 dogs with SEE.

	**All dogs (41)**	**Conservatively treated dogs (24)**	**Surgically treated dogs (17)**	***P*-value**
Hyperthermia	11 (26.8%)	5 (20.8%)	6 (35.3%)	0.476
Spinal hyperaesthesia (identified on neurologic examination)	38 (92.7%)	23 (95.8%)	15 (88.2%)	0.560
Neurological grade				0.058
0	0 (0.0%)	0 (0.0%)	0 (0.0%)	
1	7 (17.1%)	5 (20.8%)	2 (11.8%)	
2	20 (48.8%)	14 (58.3%)	6 (35.3%)	
3	11 (26.8%)	4 (16.7%)	7 (41.2%)	
4	3 (7.3%)	1 (4.2%)	2 (11.8%)	
5	0 (0.0%)	0 (0.0%)	0 (0.0%)	
Ambulatory	27 (65.9%)	19 (79.2%)	8 (47.1%)	0.048
Urinary incontinence	3 (7.3%)	1 (4.4%)	2 (11.8%)	0.565
Fecal incontinence	2 (4.9%)	1 (4.4%)	1 (5.9%)	1.000

Table 1 Clinical signs detected at the time of presentation to the referral hospital of 41 dogs with SEE.

Concurrent conditions detected on presentation to the referral hospitals included (in the 24 conservatively treated and 17 dogs who underwent spinal surgery, respectively) bilateral otitis externa (1/24 and 1/17 dogs), a heart murmur (2/24 and 1/17 dogs), tense abdomen (2/24 and 2/17 dogs), orthopedic signs (5/24 and 3/17 dogs), respiratory distress (0/24 and 1/17), and anal sac abscessation (0/24 and 1/17).

Two dogs in the surgical treatment group had undergone spinal surgery for intervertebral disc herniation prior to development of SEE; one dog developed lumbosacral SEE 749 days following cervical surgery and the other developed SEE at the thoracolumbar surgical site 15 days postoperatively.

The median time from the onset of clinical signs to SEE diagnosis was 12.5 days for all dogs (range 1–76 days), 16 days in conservatively treated dogs, and 11 days in surgically treated dogs.

Of the 41 dogs, only 2 dogs were tetraparetic (grades 2 and 3), 29 dogs were paraparetic (grade 2 in 19 dogs and grade 3 in 10 dogs), 3 dogs were paraplegic (grade 4), and 7 dogs had pain only (grade 1).

### Laboratory Findings

Anemia was present in 9/34 (26.5%) dogs that underwent hematology, characterized as mild in 8 dogs and moderate in one other dog. The mean leukocyte count was 17.3 × 10^9^/L (median 17.7 × 10^9^/L, range: 4.5–31.8 × 10^9^/L), reference interval 6.0–18.0 × 10^9^, for all dogs with SEE. Neutrophilia was observed in 19/32 (59.4%) dogs with SEE. A left shift was present in 4/32 dogs. Serum biochemistry panels revealed no significant abnormalities in any dog.

Urine culture was performed in 16/41 dogs and was positive in 2/16 (12.5%) dogs. Pre-referral antibiotic therapy had been administered in 5/16 dogs. The urine culture results were positive for *Pasteurella* spp. in one dog and both *Streptococcus canis* and *Staphylococcus pseudointermedius* in the other dog. Neither dog had received pre-referral antibiotic therapy. Blood cultures were performed in 11/41 dogs. The blood cultures were positive in 7/11 (63.6%) dogs with SEE; six were coagulase-positive *Staphylococcus* spp. and one was *Pasteurella* spp. One of the seven dogs with a positive blood culture had been administered antibiotics prior to referral. Of the aforementioned dogs, eight dogs had both urine and blood cultures performed; both cultures were negative in 3/8 dogs (37.5%).

CSF was collected in 10/41 dogs (four lumbar collection, two cisternal, one both lumbar and cisternal, and three undocumented). Analysis revealed neutrophilic pleocytosis in 4/11 samples, proteinosis in 4/11 CSF samples, and/or the presence of bacteria in 2/11 dogs (both a mixture of bacilli and cocci). CSF culture was positive in 2/6 (33.3%) dogs with SEE, with *Pasteurella* spp. found in one dog (with *Pasteurella* spp. also grown in the urine and blood) and non-hemolytic *Streptococcus* spp. found in another dog (urine culture was not performed, blood culture was negative). Neither of these dogs had received pre-referral antibiotic treatment.

Histopathological analysis of abnormal epidural material was performed in 15/19 (78.9%) surgically treated cases and confirmed SEE. Cytological examination of epidural material was performed in 12/19 surgically treated dogs, with bacteria observed in 2/12 cases. In 13/19 dogs, purulent epidural material was cultured. Eight of these dogs had received antibiotics. Bacterial growth was observed in 4/13 (30.7%) cultures; 1/8 (12.5%) dogs that had received antibiotics and 3/5 (60.0%) dogs that had not. The following bacteria were identified (one case each): *Enterobacter cloacae*, coagulase-positive *Staphylococcus* spp., coagulase-negative *Staphylococcus* spp., *Salmonella* spp., and *Pseudomonas aeruginosa*. Only one dog had blood, urine, and epidural material cultures all performed, with *Escherichia coli* and *Salmonella* spp. found epidurally and negative urine and blood cultures.

No significant differences in laboratory findings were observed between the two groups (Table 2).

**Table 2:** Laboratory findings of dogs treated conservatively and surgically for SEE at the time of admission to the referral hospital.

	**All dogs**	**Conservatively treated**	**Surgically treated**	***P*-value**
Anemia	9/34 (26.5%)	6/19 (31.6%)	3/15 (20%)	0.697
Mean leukocyte count ( × 10^9^/L)	17.7	16.5	18.3	0.425
Positive urine culture	2/16 (12.5%)	2/15 (13.3%)	0/1 (0.0%)	1.000
Positive blood culture	7/11 (63.6%)	5/8 (62.5%)	2/3 (66.7%)	1.000

### MRI Findings

The sites affected by SEE and the abnormalities detected on MRI are summarized in Table 3. Of the 7 dogs with SEE in more than one spinal region, 6/7 had SEE in the T3–L3 and L4–S3 regions and one dog, with multifocal spinal localization, had SEE at C1–T2, T3–L3, and L4–S3. Intravenous gadolinium was administered to 23/24 conservatively treated and 13/17 surgically treated dogs. Contrast enhancement of epidural material was noted in 35/36 dogs (97.2%). Of the dogs with SEE contrast enhancement, the pattern was uniform/slightly heterogeneous in 24/36 (66.7%) dogs (17/23 conservatively treated and 7/13 surgically treated dogs) and peripheral in 14/36 (39.0%) dogs (9/23 conservatively treated and 5/13 surgically treated dogs).

**Table 3:** Location and MRI findings of SEE.

	**All dogs**	**Conservatively treated dogs**	**Surgically treated dogs**	***P*-value**
Location of SEE				
Cervical (C1–C7)	2/41 (4.9%)	1/24 (4.2%)	1/17 (5.9%)	
Thoracic (T1–T13)	22/41 (80.5%)	15/24 (62.5%)	7/17 (41.2%)	
Lumbosacral	32/41 (45%)	8/24 (33.3%)	14/17 (82.4%)	Not
(L4–S3 and				performed
cauda equina)				
Concurrent discospondylitis	22/41 (53.7%)	17/24 (70.8%)	5/17 (29.4%)	0.012
Concurrent paraspinal inflammation	33/41 (80.5%)	22/24 (91.7%)	11/16 (68.8%)	0.094
Paraspinal abscessation	18/40 (45%)	9/23 (39.1%)	9/17 (52.9%)	0.523
Contrast enhancement (epidural material)	35/36 (97.2%)	23/23 (100.0%)	12/13 (92.3%)	0.406

*In some dogs, more than one spinal region was affected*.

### Treatment and Short-Term Outcome

#### Extraspinal Abscess Management

In addition to the SEE, 18 dogs presented with an extraspinal abscess, of which 15 dogs underwent surgery for abscess debridement. Of these 15 dogs having surgery for an extraspinal abscess, eight dogs had an exploratory laparotomy for a sub-lumbar abscess (3 are included in the conservatively treated group and 5 in the spinal decompressive surgery group) and the remainder had the extraspinal abscess surgically explored using the same approach as for the spinal surgery required to treat the SEE. Foreign bodies were identified in 3 dogs, all of which were a grass seed located outside the vertebral canal.

#### Conservatively Treated Dogs

Antibiotics were administered to all 24 conservatively managed dogs (amoxicillin–clavulanic acid, fluoroquinolones, cephalosporins, metronidazole, and/or clindamycin in 11, 10, 9, 7, and 2 dogs, respectively). Information on the duration of antibiotic therapy was available for 21/24 conservatively treated dogs with a median of 105 days (range: 9–651 days). Analgesia was administered to all 24 dogs (opioids, NSAIDs, gabapentin, lidocaine, paracetamol, and ketamine in 20, 15, 12, 5, 5, and 3 dogs, respectively). One dog presented to the referral center already on an anti-inflammatory dose of prednisolone, and this medication was tapered off over 7 days. Information on the duration of analgesia administration was available for 21/24 conservatively treated dogs with a median of 21 days (range: 0–385 days).

Of the 24 dogs that were initially allocated to the conservative treatment group, two dogs (presenting as grade 1 and grade 2) deteriorated neurologically (both to grade 3) during hospitalization and surgical intervention was deemed necessary. These two dogs were moved into the surgical treatment group with 22 dogs remaining in the conservatively managed group. Both dogs had a good short-term outcome and were ambulatory at discharge. One of these dogs was then lost to long-term follow-up, but the other dog was reported to have a good long-term outcome.

Three dogs (one grade 2 and two grade 3) deteriorated during hospitalization despite conservative treatment and were euthanized at their owner's request prior to discharge, meaning 19/22 conservatively managed dogs survived to discharge. The duration of hospitalization for these remaining 19 dogs managed conservatively ranged from 1 to 17 days (median 5 days), and the neurological status at discharge is presented in Table 4.

**Table 4:** Neurological status at discharge and long-term outcome of dogs treated conservatively and surgically for SEE.

	**All dogs**	**Conservatively treated dogs**	**Surgically treated dogs**	***P*-value**
Survived to discharge	37/41 (90.2%)	19/22 (86.4%)	18/19 (94.7%)	0.610
Neurological grade at discharge				
0	4/37 (10.8%)	3/19 (15.8%)	1/18 (5.6%)	0.221
1	9/37 (24.3%)	5/19 (26.3%)	4/18 (22.2%)	
2	18/37 (48.6%)	9/19 (47.4%)	9/18 (50.0%)	
3	5/37 (13.5%)	2/19 (10.5%)	3/18 (16.7%)	
4	1/37 (2.7%)	0/19 (0.0%)	1/18 (5.6%)	
5	0/37 (0%)	0/19 (0.0%)	0/18 (0.0%)	
Ambulatory at discharge	31/37 (83.8%)	17/19 (89.5%)	14/18 (77.8%)	0.405
Urinary/fecal incontinence	0/36 (0%)	0/19 (0.0%)	0/18 (0.0%)	1.000

Two dogs received physiotherapy in a physiotherapy center for <1–3 months, starting within 2 weeks of SEE diagnosis. One of these dogs also received hydrotherapy.

#### Surgically Treated Dogs (i.e., Dogs That Underwent Spinal Surgery for SEE)

Two dogs undergoing conservative management deteriorated neurologically and surgery was performed. These dogs are therefore included in the surgical group, meaning the total number of surgically treated dogs is 19 as indicated in Table 4 and the following descriptive statistics. Surgically treated dogs underwent minihemilaminectomy/hemilaminectomy (9/19) or dorsal laminectomy (10/19). Five of these dogs had a concurrent exploratory laparotomy to address an extraspinal abscess. No dog required vertebral column stabilization. A grass seed foreign body, external to the vertebral canal, was identified intraoperatively in 3/19 dogs. Sixteen dogs underwent spinal surgery <24 h after diagnosis.

Antimicrobials were administered to all 19 dogs (amoxicillin–clavulanic acid, metronidazole, cephalosporins, fluoroquinolones, clindamycin, and/or doxycycline in 11, 9, 9, 7, 3, and 1 dog, respectively) with a mean duration of 72 days (range: 11–270 days). Antimicrobial treatment was based on culture and sensitivity in 7 dogs (based on blood cultures in 5 dogs, urinary culture in 1 dog, and both urinary/blood culture in 1 dog).

Analgesia was administered to all 19 dogs (opioids, NSAIDs, gabapentin, ketamine, lidocaine, glucocorticoids, paracetamol, and/or diazepam in 19, 15, 9, 7, 5, 4, 1, and 1 dog, respectively) for a median of 42 days (range: 6–79 days).

One dog, which presented non-ambulatory paraparetic (grade 3), did not improve and was euthanized during hospitalization. The duration of hospitalization for the remaining 18 dogs ranged from 4 to 23 days (median 8 days). The neurological status at discharge was available for all 18 dogs that survived to discharge (Table 4). Urinary incontinence had resolved in the dog that presented with this.

#### Comparison Between Conservatively and Surgically Treated Dogs

There were no significant differences between the treatment groups with respect to the survival to discharge, neurological grade at discharge, or presence of urinary/fecal incontinence. The hospitalization period for surgically treated dogs was longer than for those conservatively treated and more surgically treated dogs were non-ambulatory on presentation than the conservatively managed group.

### Long-Term Outcome

Three dogs were lost to long-term follow-up in each group (six dogs in total) meaning that long-term outcome information was available for 19/22 dogs in the conservatively treated group and 16/19 dogs in the surgically treated group. The long-term follow-up ranged from 12 to 90 months (median 40 months) for conservatively treated dogs and 6–110 months (median 56 months) for surgically treated dogs.

There was no significant difference (*p* = 1.000) in long-term outcome between the conservatively and surgically treated dogs with a good long-term outcome in 15/19 (78.9%) conservatively treated and 12/16 (75%) surgically treated dogs. There was also no significant difference (*p* = 1.000) in time from onset of clinical signs to SEE diagnosis between dogs with a poor long-term outcome (onset to diagnosis mean time of 20.1 days) and dogs with a good long-term outcome (onset to diagnosis mean time of 19.4 days). Of the 7 dogs that had multiple regions affected by SEE (5 surgically treated and 2 conservatively treated), one was lost to follow-up and the other six dogs had a good long-term outcome. Mean leukocyte count, age, and ambulation status at presentation were compared between the good and poor long-term outcome groups to see if there were any significant differences. Results are summarized in Table 5.

**Table 5:** Mean leucocyte count, age, and ambulation status in dogs with good and poor long-term outcome.

	**Good long-term outcome (27 dogs)**	**Poor long-term outcome (8 dogs)**	***P*-value**
Mean leukocyte count ( × 10^9^/L)	17	19.3	0.527
Mean age (years)	5.54	8.19	0.039
Ambulation status at presentation			
Non-ambulatory	8/13 (61.5%)	5/13 (38.4%)	0.116
Ambulatory	19/22 (86.3%)	3/22 (13.6%)	

Dogs with a poor long-term outcome were significantly older (*p* = 0.039) at presentation to the referral center than dogs with a good long-term outcome. Dogs with a successful long-term outcome had a mean age of 5.5 years (median 6.46, range: 0.3–11) and a mean leukocyte count of 17 × 10^9^/L (median 17.5 × 10^9^/L, range: 4.5–31.8 × 10^9^/L), whereas dogs with a poor long-term outcome had a mean age of 8.19 years (median 8.25, range: 0.7–13.25) and a mean leukocyte count of 19.3 × 10^9^/L (median 19.8 × 10^9^/L, range: 13.3–24.5 × 10^9^/L). Mean leukocyte count was not significantly associated with a poorer outcome and the number of dogs who had blood culture was too low to include this in the statistical analysis.

## Discussion

To the authors' knowledge, this study represents the largest investigation of canine SEE conducted to date.

### Signalment and Clinical Presentation

In our study population, male dogs with SEE were overrepresented (63.4%), with a higher proportion of entire male dogs [16/26 (61.5%)] than entire female dogs [5/19 (26.3%)]. In previous studies on SEE in dogs, there was no apparent age, gender, or breed predisposition (5, 6).

Spinal hyperesthesia was a very common presenting finding (38/41 dogs, 92.7%), in agreement with previous reports in both animals and humans (4–6, 16, 29). Pyrexia at presentation was not common (11/41 dogs, 26.8%). The incidence of pyrexia is less common in our canine population than in the two large human studies, which reported fever in 37–66% of patients with SEE (28, 29).

Surgically treated dogs were significantly more likely to present non-ambulatory (*p* = 0.048), suggesting that the attending clinicians may have been more inclined to treat surgically non-ambulatory than ambulatory dogs. The extent of the empyema and financial considerations may also have influenced the decision to treat surgically or conservatively.

### Laboratory Findings

Positive identification with culture and sensitivity is preferable for antibiotic stewardship and for optimizing outcomes in bacterial infections. In this study 15/41 dogs had received pre-referral antibiotic treatment. There was a higher percentage of positive blood cultures (7/11, 63.6%) than urine cultures (2/16, 12.5%); however, a higher percentage of dogs in the urine culture group had already received pre-culture antibiotics (5/16, 31%) compared with the blood culture group (1/11, 9%). This difference in pre-referral antibiotic treatment may explain the difference in positive culture rates. Over half (4/7) of the dogs with positive blood cultures had a concurrent urine culture and 3/4 of these urine cultures were negative. One dog had the same bacteria identified in both urine and blood cultures. Of the two dogs with positive urine cultures, one dog had the same bacteria (*Pasteurella* spp.) identified on blood culture and the other did not have a blood culture performed. The sensitivity of blood and urine cultures for identification of causal organisms in SEE requires further investigation, but our findings suggest that blood culture may be beneficial despite a negative urine culture.

### MRI Findings

SEE was more common in the lumbosacral (32/41 dogs, 78%) and thoracic vertebral column (22/41 dogs, 53.7%) than the cervical vertebral column (2/41, 4.9%). A possible explanation for the skew toward the lumbosacral vertebral column may be the proximity of the genitourinary tract, mobility within the L7/S1 disc space, and the lumbar region being a common place for sublumbar abscesses and foreign bodies. The L7–S1 intervertebral disc space seems to be the most common individual disc site affected by discospondylitis in a study of 513 dogs with discospondylitis (45).

Concurrent discospondylitis was common in our study population (53.7% of all dogs). This is similar to previous studies of SEE in dogs (5, 6). Discospondylitis is present in 95% of human patients with SEE (46). Discospondylitis was identified significantly (*p* = 0.012) more frequently in conservatively managed (17/24 dogs, 70.8%) than surgically managed cases (5/17, 29.4%). It is possible that the presence of discospondylitis was a negative factor in the decision-making process for surgery. The clinician may feel that discospondylitis could lead to more chance of instability following spinal surgery. This could lead them to pursue conservative management preferentially in these cases as the use of implants with an active bacterial infection is more prone to complications.

Contrast enhancement of the epidural material was very common in the 36/41 SEE cases that received contrast (35/36 dogs, 97.2%), which would be expected with the pathophysiology of the disease process and is consistent with findings in a previous study on MRI findings in SEE in dogs (6). The contrast enhancement pattern in our study was uniform/slightly heterogeneous in 24/36 (66.7%) dogs and peripheral in 14/36 (39.0%) dogs. The presence of two distinct contrast patterns (both diffuse and peripheral) in our study is similar to that described previously in a paper on MRI findings in five dogs with SEE (6).

### Outcome

Despite greater clinical severity at presentation in surgically treated dogs, there was no significant difference in long-term outcome between conservatively and surgically treated dogs (78.9 and 75.0%, respectively). Our study suggests that conservative treatment may be appropriate for dogs with SEE that are ambulatory, provided there is no clinical progression during conservative management. These findings are clinically important because not every animal is a suitable surgical/anesthetic candidate and not every owner is prepared or financially able to pursue surgical treatment.

Most SEE studies in people also fail to demonstrate a significant difference in outcome between conservatively and surgically treated patients (1, 27, 33, 37, 47–51). However, some studies do not comment on the patients' pre-treatment neurological status (47–50), and in most studies, as in ours, surgically treated patients exhibit more severe pre-treatment neurological signs compared with conservatively treated patients (27, 33, 37, 51). Only Curry et al. (30) demonstrated a significant difference in outcome between conservatively and surgically managed patients, with the latter exhibiting a better outcome (13 vs. 60%) (30). Failure of conservative treatment and the subsequent need for surgery has been reported in 9.5–47.8% of human cases (27, 30, 33, 37, 47).

Risk factors for failure of conservative management in humans include diabetes mellitus, a C-reactive protein (CRP) >115 mg/L, a leukocyte count >12 × 10^9^/L, positive blood cultures, age >65 years, the presence of methicillin-resistant *Staphylococcus aureus*, and advanced neurological deficits (33, 39, 52). No such information exists in the veterinary literature.

As we only had two dogs in this study that failed conservative management, we looked for differences between the good and poor long-term outcome groups with respect to leukocyte count, positive blood culture, age, and neurological status at presentation. Positive blood cultures could not be evaluated statistically due to the small number of dogs (*n* = 8) having blood culture performed. There were no significant differences between the long-term outcome groups for leukocyte count or neurological status at presentation; however, dogs with a poor long-term outcome were significantly older. Only eight dogs had a poor outcome in our study, which meant that the statistical power in the outcome analysis was low. The percentage of dogs with a poor long-term outcome that presented non-ambulatory or ambulatory is quite different between the groups, and although we did not achieve statistical significance (*p* = 0.116), this may be a factor that does show significance with a larger sample size. A prospective study with larger case numbers looking at risk factors for poor outcome including CRP, leukocyte count, blood culture, age, and severity of neurological deficits would be indicated in the future to further help in the decision-making process of which dogs should be managed surgically compared with conservatively.

Of the dogs that survived to discharge, most were ambulatory (31/37) at the time of discharge. For the dogs (6/37) that were non-ambulatory at discharge, the exact time to regain ambulation is known in three dogs with a median of 7 days and a mean of 15.75 days (range 7–28 days). All but two dogs with a successful long-term outcome were neurologically normal at the time of data collection. Thus, our study suggests that most dogs that become neurologically normal with a successful long-term outcome are functional pets within a month of treatment initiation.

### Limitations

The retrospective design of the study limited the investigation, with biases for data collection and variability in the evaluation of objective clinical outcome measures. A direct comparison of outcomes between the treatment groups must be interpreted cautiously due to the differences in presentation. A more severe neurological grading increased the likelihood of surgical treatment; therefore, the outcome in non-ambulatory dogs receiving conservative treatment is unknown. A prospective study would help to test this aspect more reliably; however, it can be argued that it would be unethical not to provide spinal decompression to dogs with severe neurologic dysfunction when their owners are willing to pursue surgery. Furthermore, the study population was composed of five referral hospital caseloads, potentially introducing bias toward more severe clinical phenotypes and/or owners with fewer financial constraints than the general population. Although larger studies, permitting multivariate analyses, would be very valuable, SEE is a relatively rare condition and obtaining sufficient case numbers is problematic.

Not all clinical presentation, diagnostic, therapeutic, and outcome variables were available for all dogs. As these missing data may have affected the results obtained, this was also a study limitation.

Two dogs failed conservative management (becoming non-ambulatory after initially presenting with neurological grades 1 and 3) and were subsequently treated surgically. Unfortunately, case numbers in this study were insufficient to create a third group, namely, failed conservative management requiring surgery.

Larger prospective studies are required to identify prognostic factors associated with recurrence, treatment failure, and poor outcome in each treatment group.

### Conclusions

Our data revealed no significant difference (*p* = 1.00) in long-term success rates between conservatively and surgically managed dogs; however, significantly more cases within the surgically treated group were non-ambulatory on presentation. This study suggests that conservative treatment may be appropriate for dogs with SEE that are ambulatory at presentation, although a further study evaluating whether dogs that fail conservative management and subsequently undergo spinal surgery also have good long-term outcomes would be indicated.

Despite the limitations of our study, it remains the largest study on spinal epidural empyema to date and the results can help in clinical decision-making and inform discussion with owners of dogs diagnosed with SEE. Further studies are needed to compare conservative and surgical treatment of SEE in a population of dogs with similar clinical presentations to determine prognostic indicators of treatment response and indicators of disease recurrence.

## Data Availability Statement

The raw data supporting the conclusions of this article will be made available by the authors, without undue reservation.

## Ethics Statement

The animal study was reviewed and approved by Animal Health Trust Clinical Research Ethics Committee. Written informed consent was obtained from the owners for the participation of their animals in this study.

## Author Contributions

EL and LD: study design, data collection, data analysis, manuscript writing, and manuscript revision. LS: study design, data collection, manuscript writing, and manuscript revision. EB and JB: data collection and manuscript revision. ED: data collection, data analysis, and manuscript revision. AE: manuscript revision. All authors contributed to the article and approved the submitted version.

## Conflict of Interest

EL, LD, LS, and JB were employed by Linnaeus Veterinary Limited. The remaining authors declare that the research was conducted in the absence of any commercial or financial relationships that could be construed as a potential conflict of interest.

## Publisher's Note

All claims expressed in this article are solely those of the authors and do not necessarily represent those of their affiliated organizations, or those of the publisher, the editors and the reviewers. Any product that may be evaluated in this article, or claim that may be made by its manufacturer, is not guaranteed or endorsed by the publisher.
